# Programmable mechanical metastructures modeling polydomain materials

**DOI:** 10.1126/sciadv.adz9811

**Published:** 2025-10-03

**Authors:** Yifan Yang, Xiaoliang Zhang, Ting Wang, Xinqi Lin, Fan Xu

**Affiliations:** ^1^ Institute of Mechanics and Computational Engineering, Department of Aeronautics and Astronautics, College of Intelligent Robotics and Advanced Manufacturing, Fudan University, Shanghai 200433, P.R. China.; ^2^ International Institute for Intelligent Nanorobots and Nanosystems, College of Intelligent Robotics and Advanced Manufacturing, Fudan University, Shanghai 200433, P.R. China.; ^3^ School of Aerospace Engineering and Applied Mechanics, Tongji University, Shanghai 200092, P.R. China.; ^4^ Shanghai Innovation Institute, Shanghai 200231, P.R. China.; ^5^ State Key Laboratory of Coatings for Advanced Equipment, Fudan University, Shanghai 200433, P.R. China.

## Abstract

The orientation and distribution of microscopic units in polydomain materials contribute to their functionality and performance. However, within bulk materials from traditional synthesis and modeling, achieving flexible and precise arrangements of the microscopic units is challenging, which restricts the capabilities to fully understand and manipulate their polydomain-based properties. Here, we present a metastructure to mimic both mesoscale phase change and macroscopic mechanical properties of polydomain materials such as liquid crystal elastomers, which achieves unprecedented tunability over domain structures through the rational design of unit cells and their spatial arrangements. These metastructure system is used to explore and directly visualize the complex mesoscale topological deformation mechanisms hidden at molecular scales, providing fundamental insights into the mechanical properties of these materials. Beyond mimicking known polydomain materials, we demonstrate functionalities including mechanical encoding/decoding and programmable shape morphing. Our results establish a framework for understanding and designing topology-tunable functional polydomain materials.

## Teaser

Metastructures with programmable domain arrangements mimic polydomain materials, enabling mechanical encoding and shape morphing.

## INTRODUCTION

Many materials exhibit polydomain microstructures, which widely exist in atomic crystals (*1*), magnetic films (*2*), and liquid crystal elastomers (*3*). The domain distribution and arrangement in these materials are closely correlated with their properties (*4*–*8*), and the regulation of the domain distribution plays a crucial role in functional design and performance enhancement (*3*, *9*–*11*). For example, to design and fabricate polydomain liquid crystal elastomers (LCEs) with optimal performance, which are widely used in soft actuators (*12*, *13*) and energy dissipation systems (*14*), it is essential to precisely control the director arrangement (*15*, *16*). Conventional bulk materials obtained from chemical synthesis (*17*–*19*) often struggle to achieve precise tunability at the single-molecule level.

The microstructures of classical LCEs comprise relatively stiff rod molecules and flexible polymer chains (*10*). Under external stimuli such as light (*20*, *21*), heat (*22*–*24*), electromagnetic field (*25*, *26*), and mechanical force (*27*, *28*), the rod molecules undergo rigid-body displacement and rotation, while the flexible chains experience stretching deformation. This coupling of motion modes, in conjunction with specific molecular responses to external fields, underpins the functionality of various LCEs (*29*). While this flexible chain and rigid rod crosslinking structure can be described using statistically based network theory (*30*, *31*), this theory cannot accurately analyze the effects caused by rigid rod rotation within the microstructure, limiting the understanding of the microscopic mechanisms underlying the unique behaviors of LCEs.

Here, inspired by polydomain materials, we design a metamaterial system, with lattice domains comprised of thermoplastic polyurethane (TPU) and polylactic acid (PLA), to mimic the multiscale anomalous behavior of LCEs.

We show that, both experimentally and theoretically, the metamaterial lattice sheets can effectively mimic the anomalous shear deformation, polydomain-monodomain transition, and soft elasticity (unique plateau-like response) experienced by nematic LCE films upon stretching. This macroscopic metamaterial can perfectly emulate the microscopic changes in the orientational order of LCEs, which can be used as a platform to explore and visualize domain-assisted deformation mechanisms, bridging molecule-level configurations with macroscopic properties. Beyond conventional polydomain materials, the presented metamaterial enables precise control at the lattice scale, endowing the structure with a higher design ceiling to achieve topology-determined functions and applications. As examples, we demonstrate a counterintuitive strip exhibiting zigzag bending when stretched, mechanical encoders, and decoders by leveraging the complex domain-based nonlinear deformations.

## RESULTS

### Model

**Fig. 1.**
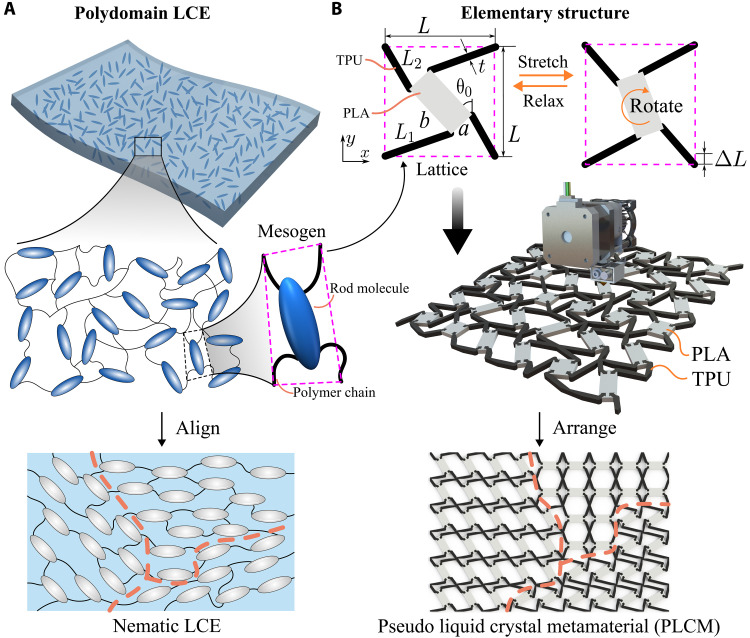
Schematic of polydomain LCE. (**A**) Molecular-level organization of LCE units composed of rod molecules and polymer chains and their alignment for polydomain distribution. (**B**) Design of pseudo liquid crystal metamaterial (PLCM) mimicking LCE microstructure and their polydomain arrangement.

We develop a homogenized model based on micropolar theory to predict the anomalous mechanical behaviors of polydomain soft materials such as LCEs. The microscopic rotational deformation plays a crucial role in the unique behaviors in polydomain soft materials. To accurately characterize this behavior, we apply a micropolar theory, which introduces a rotational deformation $\phi_{i}$ (*32*) of a square lattice composed of four soft beams and a rigid block, as shown in Fig. 1. Compared to higher-order homogenized models (*33*–*35*) that consider higher-order displacement fields and focus on strain gradient effects, the micropolar theory adopted here explicitly introduces rotational degrees of freedom, providing a natural framework for describing the rotational behavior of material microstructures. The symbols a and b denote the side lengths of the rectangular block, while $\theta_{0}$ represents the initial direction of the block. It can be observed that the initial direction of the block dictates the chirality of the lattice. An opposite $\theta_{0}$ leads to an opposite chirality, and when $\theta_{0}=0$, the chirality of the structure disappears. The geometric and equilibrium equations of a planar micropolar elastomer read $\varepsilon_{\alpha \beta }=u_{\beta ,\alpha }+e_{3\beta \alpha }\phi_{3},\kappa_{\alpha 3}=\phi_{3,\alpha }$ and $\sigma_{\beta \alpha ,\beta }=\rho \;\partial^{2}u_{\alpha }/\partial t^{2},m_{\alpha 3,\alpha }+e_{3\alpha \beta }\sigma_{\alpha \beta }=J\;\partial^{2}\phi_{3}/\;\partial t^{2}$, where $\varepsilon_{\alpha \beta }$ and $\sigma_{\alpha \beta }$ represent, respectively, the strain and stress, $\kappa_{\alpha 3}$ denotes the curvature, $m_{\alpha 3}$ is the couple stress, ρ is the density, J is the micro-inertia, and $e_{3\alpha \beta }$ can be considered as a two-dimensional (2D) Levi-Civita tensor. Greek indices α,β,… take values in {1, 2}, while Latin indices i,j,… run from 1 to 3. A comma in subscript denotes a partial derivative and we use Einstein’s convention for implicit summation on repeated indices. The constitutive equations can thus be expressed as $\sigma_{\alpha \beta }=C_{\alpha \beta \gamma \rho }\varepsilon_{\gamma \rho }+H_{\alpha \beta \rho }\kappa_{\rho 3},m_{\rho 3}=H_{\alpha \beta \rho }\varepsilon_{\alpha \beta }+D_{\rho \gamma }\kappa_{\gamma 3}$, where $C_{\alpha \beta \gamma \rho }$, $H_{\alpha \beta \rho }$, and $D_{\rho \gamma }$ represent elastic tensors. Therefore, the strain energy density can be given by

$$\psi =\frac{1}{2}C_{\alpha \beta \gamma \rho }\varepsilon_{\alpha \beta }\varepsilon_{\gamma \rho }+\frac{1}{2}D_{\alpha \beta }\kappa_{\alpha 3}\kappa_{\beta 3}+\varepsilon_{\alpha \beta }H_{\alpha \beta \gamma }\kappa_{\gamma 3}$$ (1)

Liu *et al.* (*36*) proved that due to the incompatibility with 2D situations, the third-order tensor H must vanish. Therefore, the constitutive laws can be simplified as $\sigma_{\alpha \beta }=C_{\alpha \beta \gamma \rho }\varepsilon_{\gamma \rho },m_{\rho 3}=D_{\rho \gamma }\kappa_{\gamma 3}$.

We use the strain energy density for homogenization and use $u_{p,q}={\left[u_{0}\;v_{0}\;\phi_{0}\right]}^{T}$ to denote the motion of the bottom left corner in the lattice marked as (p,q) (see fig. S1). The strain energy density of lattice (p,q) can then be determined as $\psi_{p,q}=\psi \left(u_{p,q},u_{p+1,q},u_{p,q+1},u_{p+1,q+1}\right)$. Because the rectangular block is assumed to be a rigid body, only the strain energy of four soft beams needs to be considered, which can be obtained on the basis of the Euler-Bernoulli beam theory

$$\left\{\begin{array}{c} W_{1}=\frac{1}{2}{\left\{u_{p,q}\;u_{A}\right\}}^{T}{K|}_{l=L_{1}}\left\{u_{p,q}\;u_{A}\right\}\\ W_{2}=\frac{1}{2}{\left\{u_{p,q+1}\;u_{A}\right\}}^{T}{K|}_{l=L_{2}}\left\{u_{p,q+1}\;u_{A}\right\} \end{array}\right.$$ (2)

where $u_{A}$ denotes the displacement and rotation degrees of freedom associated with the center of the rigid block and K is the stiffness matrix of the soft beams. Because of symmetry, the strain energy density of the lattice (p,q) is eventually given by

$$\psi_{p,q}=2\left(W_{1}+W_{2}\right)/L^{2}$$ (3)

At this point, by performing a partial derivative calculation on the energy density, the material parameters can be straightforwardly obtained.

### Mimicking inhomogeneous deformations of LCEs

We first design metamaterial sheets to mimic the unique behaviors of LCE films. We use 3D printing technology that facilitates programmable fabrication of metamaterial sheets with precise microstructure arrangement. As mentioned above, the lattice comprises two parts, i.e., soft beams and a rigid block. Two common and cost-effective materials, PLA and TPU, are used as inks in the 3D printing. We apply a dual-extruder fused deposition modeling 3D printer for the simultaneous printing of both materials. Driven by the notable difference in the elastic modulus of these two materials ($E_{\text{PLA}}\approx 2600$ MPa and $E_{\text{TPU}}\approx 9.5$ MPa), blocks made of PLA remain almost undeformed even when the beams made of TPU undergo notable deformations. This feature constitutes the core design of the lattice.

**Fig. 2.**
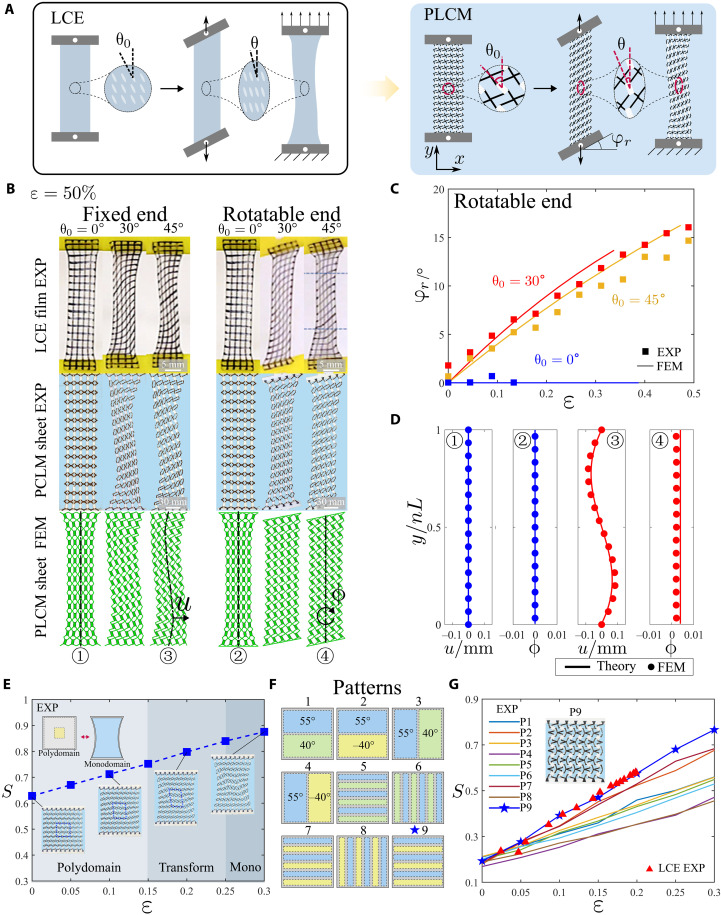
Uniaxial stretching and P-M transitions of PLCM sheets. (**A**) Stretch-induced shear deformations of LCE films and PLCM sheets. (**B**) Comparison on stretch-induced deformations of LCE films (*37*) and PLCM sheets with different initial orientations θ_0_ and boundary conditions. (**C**) Evolution of end rotation angle with increasing strain in experiments and simulations. (**D**) Comparison of theory and FEM at ε = 0.5% under different boundary conditions. (**E**) Evolution of order parameter S in PLCM sheets upon stretching. (**F**) PLCM sheets with diverse ploydomain arrangements mimicking P-M transition characteristics in LCEs. (**G**) Order parameter variations of different polydomain arrangements compared to the real LCE (*41*).

Nematic LCE films upon uniaxial stretch exhibit unusual shear deformations (*37*), as shown in Fig. 2A. When the direction of the tensile force differs from the initial orientation of the mesogens in the LCE film (i.e., θ_0_ ≠ 0°), highly inhomogeneous shearing deformation occurs. However, if the fixture allows the ends to rotate freely, the deformation turns out to be nearly homogeneous. Here, we mimic the unique deformation behaviors of LCE film by metamaterial sheets. The cellular lattice parameters are set as L=15 mm, $\mathrm{ab}\;/\;L^{2}=0.225$, η=b/a=2.5, and t=L/10. The lattices are arranged in a 15 by 5 configuration to imitate the LCE films in He *et al.* (*37*). As illustrated in Fig. 2B, three different sheets consisting of lattices with θ_0_ = 0°, 30°, and 45° are printed and stretched, respectively. Besides, finite element simulations are performed. Figure 2B demonstrates that the metamaterial sheets and nematic LCE films exhibit highly similar deformations under uniaxial stretching. Notable inhomogeneous shearing and necking deformations appear in the sheets of θ_0_ = 30° and θ_0_ = 45° with fixed ends. For rotatable boundary, when the stretching direction differs from the order direction of cellular lattice, the sheet rotates spontaneously upon tension. This rotation occurs because the stretching of the network drives the reorientation of connected units, generating torques that cause global anisotropic shear deformation. Under rotatable end conditions, the absence of rotational constraints allows both the pseudo liquid crystal metamaterial (PLCM) sheets and LCE films to minimize elastic strain energy through following the boundary rotation. The relation between the rotation angle $\varphi_{r}$ and stretching strain ε is plotted in Fig. 2C, where the experiments and simulations are highly consistent. The results suggest that our cellular lattice design highly mimics the key mechanical characteristics of LCEs.

We then apply the micropolar homogenization theory to predict unusual deformations of the metamaterial sheets. For simplicity, insignificant effect of the *x* coordinate on the deformation can be neglected (Supplementary Materials). Then, based on the constitutive and equilibrium equations, we have

$$\begin{array}{c} \left\{\begin{array}{c} 0=\left(\beta_{2}-\beta_{1}-\alpha +k+\mu \right)u^{\prime \prime }-\left(C_{1}-B-A\right)v^{\prime \prime }+\left(-\beta_{1}+\beta_{2}+2k\right)\phi^{\prime }\\ 0=-\left(C_{1}-B-A\right)u^{\prime \prime }+\left(\alpha -\beta_{1}-\beta_{2}+\lambda +2\mu \right)v^{\prime \prime }+\left(2A-C_{1}+C_{2}\right)\phi^{\prime }\\ 0=\left(\gamma -\gamma_{1}\right)\phi^{\prime \prime }+\left(\beta_{1}-\beta_{2}-2k\right)u^{\prime }+\left(C_{1}-C_{2}-2A\right)v^{\prime }-4k\phi \end{array}\right. \end{array}$$ (4)

where u, v, and ϕ represent the displacements in the horizontal direction, vertical direction, and rotation, respectively. The derivative here refers to the vertical direction. For the clamped-end sheets, the boundary conditions read u(0)=0, v(0)=0, ϕ(0)=0, u(nL)=0, v(nL)=Δv, and ϕ(nL)=0, where n=15, Δv=nL×0.005. When the ends can rotate freely, the boundary conditions are given by u(0)=0, v(0)=0, ${u^{\prime }}^{\prime }\left(0\right)=0$, u(nL)=0, v(nL)=Δv, and $u^{\prime \prime }\left(\mathrm{nL}\right)=0$. As shown in Fig. 2D, lateral displacements of the middle axis are extracted from FEM simulations and compared with the theory. When θ_0_ = 0°, the cellular lattice undergoes pure elongation deformations. When θ_0_ ≠ 0°, e.g., 30° or 45°, inhomogeneous deformations of the sheets appear due to the nonuniform rotation of cellular lattices in sheets with clamped ends, while uniform rotation of lattices can be observed in sheets with free ends.

LCEs are generally fabricated in a polydomain state without special treatment (*38*). The arrangement of molecules may vary in the polydomain LCEs, which exhibits isotropy at the macroscopic scale, while mechanical stretching can make polydomain LCEs reversibly transit into a monodomain state (*39*). The metamaterial sheets exhibit intriguing stretch-induced polydomain-monodomain (P-M) transition behaviors. As shown in Fig. 2E, a polydomain metamaterial sheet is composed of two types of lattice domains. When uniaxial stretching is imposed, the cellular lattices start to rotate toward the tension direction, and the two domains eventually merge into a single domain. The geometric parameters of the two lattices read L=15 mm, $\mathrm{ab}/L^{2}=0.225$, η = 2.5, t=L/10, and θ_0_ = ±30°. To quantitatively characterize the process of P-M transition, an order parameter S measuring the molecular alignment (*40*) can be defined as

$$S=\frac{1}{2N}\sum_{i=1}^{N}\left(3{\text{cos}}^{2}\theta_{i}^{\prime }-1\right)$$ (5)

where N denotes the number of cellular lattices, $\theta_{i}^{\prime }=90^{\circ }-\theta_{i}$, and θ*_i_* represents the direction of the rigid block in lattice *i* under a certain strain. When S≈0, the sheet is considered isotropic, and when S=1, the sheet is perfectly ordered. By measuring θ*_i_* of each cell, the relation between the order parameter S and stretching strain ε is plotted in Fig. 2E, where S increases and approaches to 1 with the rise of stretch, indicating that the polydomain sheet is transforming into a monodomain (movie S1).

To further demonstrate the strong programmability of the metamaterial sheet, nine polydomain lattices (patterns 1 to 8) are designed in Fig. 2F. The experiments indicate that the patterns with lateral strip domains (P1, P2, P5, and P7) have steeper S−ε curves in Fig. 2G. In addition, compared to P1 and P5, the patterns with opposite θ_0_ (P2 and P7) behave more similarly to the real LCEs. This is because opposite θ_0_ leads to an opposite rotation direction of the lattice, which further facilitates the rotation of adjacent lattices. Especially for P7, the lateral strip domains with opposite θ_0_ are arranged alternately and lead to a steeper curve, more close to the real LCEs (black triangles in Fig. 2G) (*41*). For a more precise imitation, we modify the parameter η from 2.5 to 1.5 based on P7 and obtain P9 (see the inset in Fig. 2G). The metamaterial sheet with well-designed pattern 9 perfectly mimics the P-M transition of the real LCEs under stretch.

### Mimicking soft and semisoft elasticity

**Fig. 3.**
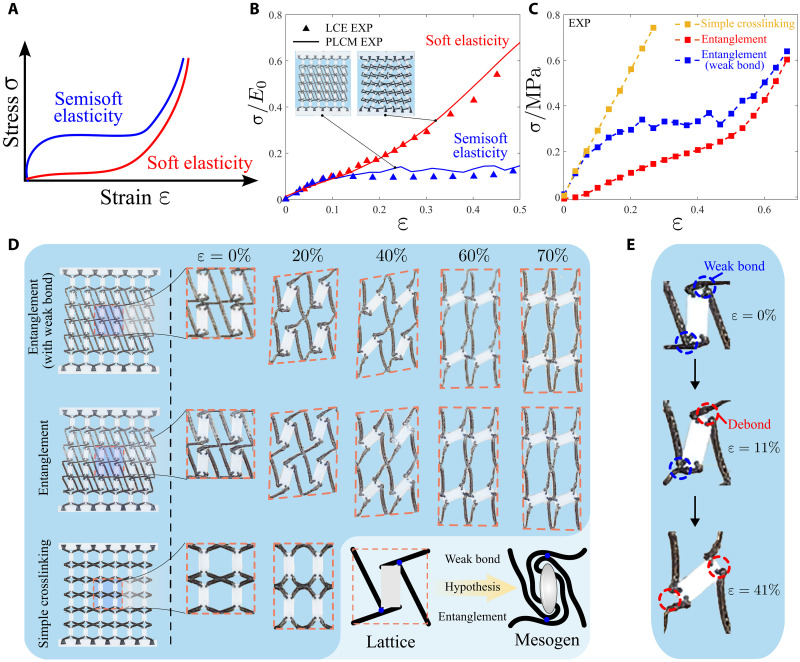
Imitation of soft/semisoft elastic responses of LCEs using PLCM. (**A**) Schematic of soft elasticity and semisoft elasticity. (**B**) Comparison of stress-strain curves between PLCM and LCE (*47*, *48*). (**C**) Stress-strain curves of three PLCM sheets with different crosslinking topologies, revealing that weak bonds produce notable plateau characteristic of semisoft elasticity. (**D**) Deformations of different PLCM sheets upon stretching. Entanglement cells enable large lattice rotation demonstrating soft/semisoft elasticity, while simple crosslinking cells result in symmetric deformation without rotation. (**E**) Debonding process of connecting beams in entanglement cells upon stretching.

Soft elasticity is a unique characteristics of LCEs, i.e., a “stress-strain plateau” upon excess stretching (*42*, *43*). Ideal soft elasticity refers to the ability of LCEs to undergo mechanical deformation without (or very low) stress (*44*) (see Fig. 3A). Because of difficulties in direct observation of LCE microstructures with clarity (*45*), there is still no consensus on the underlying mechanism on soft elasticity in LCEs (*46*). Warner and Terentjev (*10*) suspected that this characteristics may stem from the rotational behavior of the liquid crystal molecules. Here, we look into soft elasticity, aiming to provide a physical understanding on mechanism of this behavior. Experiments imply that the metamaterial can exhibit a similar soft elastic response, as shown in Fig. 3B, where the soft elastic behavior of LCE (*47*) is replicated by PLCM with polydomains. The stress-strain curves of the metamaterial and LCE are highly consistent during the stretching process.

Most LCEs, in fact, do not exhibit perfect soft elasticity (*48*) but “semisoft elasticity” due to inevitable imperfect crosslinking in LCE microstructures. Verwey and Warner (*49*) developed a statistical model to explain semisoft elasticity, yet the microscopic mechanism remains a subject of debate (*50*, *44*). We show that soft and semisoft elasticity behavior can be flexibly programmed in the pseudo liquid crystal metamaterials. As illustrated in Fig. 3 (C and D), uniaxial stretching experiments are conducted on three metamaterial sheets composed of three kinds of lattices with different crosslinking topologies. We define a simple crosslinking cell as the direct connection between the vertex of the lattice and the nearest corner of the rigid block, while the entanglement cell is characterized by the sequential connection of a lattice’s vertices to the corners of a more distant rigid block. In addition, the entanglement cell is characterized by a small aspect ratio (η<1) and a large initial orientation angle (θ_0_ = 90°) of rigid blocks. In this configuration, the short edge corresponds to the rod molecule orientation and rotates to align with the stretching direction during deformation. Meanwhile, the large initial orientation angles of rigid blocks enable the connecting beams to approach and adhere to each other sufficiently. The relatively less stable adhesions in this entanglement topology form weak bond-like interactions. It can be observed in Fig. 3D that during the stretching process, the lattices in the metamaterial sheet with entanglement cells undergo notable rotation, demonstrating soft or semisoft elasticity characteristics. Note that the connecting beams in entanglement cells gradually debond during the stretching process (Fig. 3E), releasing stress and producing a similar stress-strain plateau curve observed in the semisoft elasticity in LCEs. In contrast, lattices with a simple crosslinking cell do not rotate but experience symmetric deformation, which is consistent with the conjecture in Warner and Terentjev (*10*). Furthermore, weak bonds impede the rotational behavior of the lattices, resulting in a notable plateau in the stress-strain curve, namely, semisoft elasticity (movie S2). This rotational mechanism in the entanglement cells captures the essential mechanical responses of soft and semisoft elasticity through well-designed geometric constraints and beam interactions that mimic the role of molecular chains. The experiments remind us of the weak chemical bonds in molecular materials that are the first to break under tension. Therefore, we speculate that the soft and semisoft elastic responses exhibited by LCEs primarily originate from the rotation of rod molecules in the microstructure following entanglement crosslinking and stretching. The cause of semisoft elasticity is likely attributed to the weak chemical bonds resulting from imperfect crosslinking, which initially hinder the rotation of rod molecules, leading to a steeper stress-strain curve. After these weak chemical bonds are broken, the rod molecules rotate more freely, resulting in a plateau in the stress-strain curve of LCEs.

### Unusual zigzag deformation of stretched strips

**Fig. 4.**
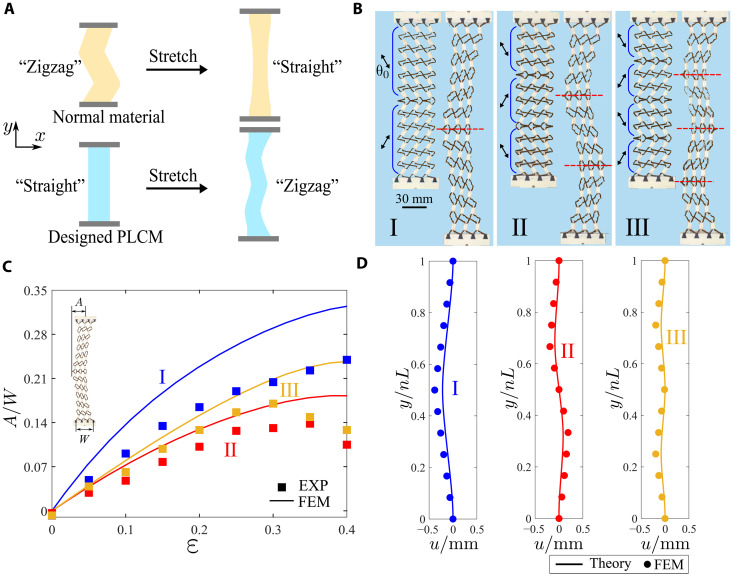
Programmable zigzag deformation design of PLCM with polydomains. (**A**) Schematic of the unusual zigzag deformation of PLCM strips under uniaxial stretching. (**B**) Experiments of zigzag deformations where domain boundaries serve as lines of inflection. (**C**) Comparisons on amplitude variations of experiments and FEM simulations. (**D**) Comparisons of theoretical prediction and FEM simulation for stretched PLCM strips (ε = 0.5%).

The well-designed PLCM metamaterials hold interesting features driven by the flexible programmability. For example, a strip with unusual deformation property can be realized by delicate arrangement of lattices. Conventional strips tend to straighten when stretched, as shown in Fig. 4A. By carefully programming polydomains, the metamaterial strips can exhibit unusual zigzag deformations upon uniaxial stretching (movie S3). The initially straight strips deform into zigzag shapes upon tension, with each boundary of adjacent domains becoming a line of inflection (see Fig. 4).

Our micropolar homogenization model can predict the unusual zigzag deformation. By homogenizing different domains and then assembling them, a set of ODEs can be obtained. Figure 4D indicates that our model accurately predicts the zigzag deformation, consistent with FEM simulations. The realization of these counterintuitive strips is attributed to the precise program of lattices and domain distribution. When combined with ingenious domain design, it endows the metamaterials with application potential.

### Smart patterning information

Combining the programmable cellular rotation and displacement of the metamaterial can carry pattern information, which implies the corresponding recoverable unique deformations can be used for storage, transformation, and encryption of patterned information. Thus, based on a thorough design of mechanical properties and deformations, we demonstrate several relevant applications.

**Fig. 5.**
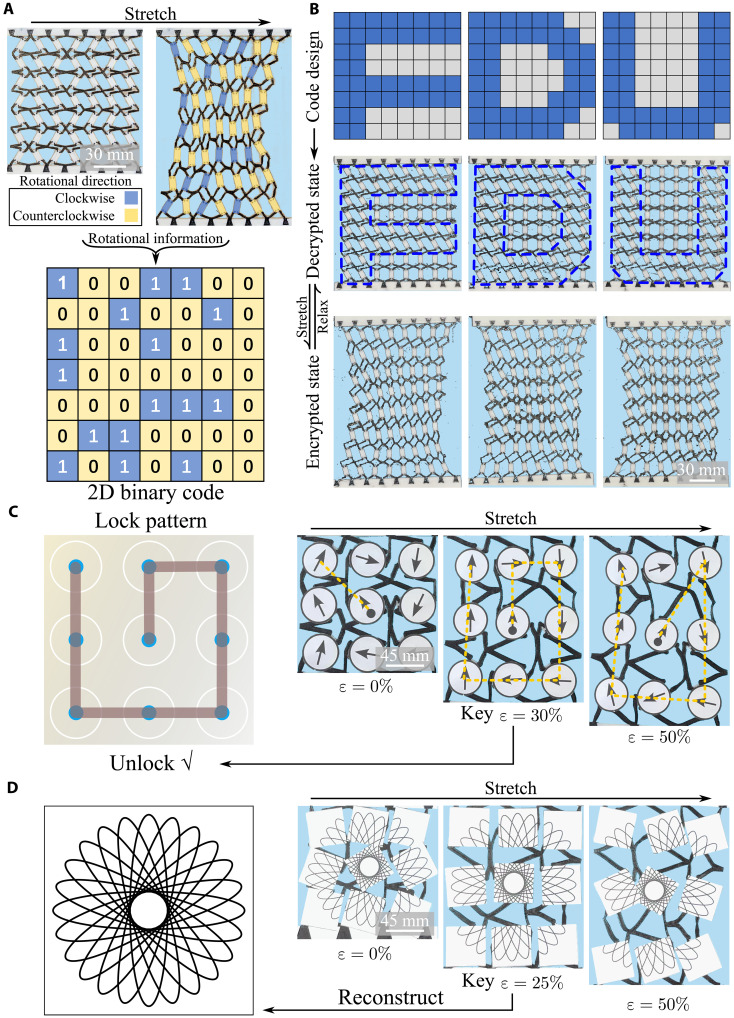
Information encoding based on polydomain metamaterial patterning. (**A**) Binary encoding based on rigid block rotation directions, i.e., clockwise for “1” and counterclockwise for “0.” (**B**) Visual pattern encryption using polydomain arrangement with FDU symbol, which becomes blurred under stretching and reappears upon load release. (**C**) Pattern lock decoder revealed at specific strain (key ε = 30%). (**D**) Strain-triggered image reconstruction shows scattered puzzle pieces assembling into a complete “geometric flower” at the key ε = 25%.

The rotation of lattices can be used for storing and transmitting information. As shown in Fig. 5A, we binary encode the rotational direction of each lattice as “1” (clockwise) or “0” (counterclockwise). By comparing the directional changes of each lattice in the metamaterial sheet before and after stretching, a 2D binary code (e.g., the widely used QR code) can be obtained. As the main characteristics of the lattice, the initial angle of the rigid block θ_0_ not only drives the rotational properties of the lattice but also has visual effects. As demonstrated in Fig. 5B, two kinds of lattices (θ_0_ = 30° and 0°) are arranged into 8×8 pixels to form the symbol “FDU” (see blue dash outlines). With the deformation differences between these two types of lattices under uniaxial stretching, the visual symbol FDU formed by the two types of lattices gradually become blurred in the deformed sheet, hiding the information. When the encrypted sheets are released, the written pattern information reappears.

Different lattices within the metamaterial exhibit distinct rotational properties and influence each other. These complex deformation characteristics can be designed to functions such as decoder for decrypting encrypted pattern information. For instance, we use nine lattices to decode a pattern lock password, as shown in Fig. 5C. We bind the starting point and directions of the pattern lock code to nine lattices using each lattice’s unique stretch-induced rotation characteristics to encrypt the pattern lock password. Then, a specific decoding strain (e.g., key ε = 30%) is set, which reveals the correct decoding method when the strain reaches this value. Different pattern lock codes appear when the decoding strain is incorrect to prevent brute-force decoding. Similarly, this decoding strategy can be applied to graphical puzzle decoding. As illustrated in Fig. 5D, an image of “geometric flower” is divided into nine pieces and bound to a piece of metamaterial sheet. Under most strains, the nine pieces of the puzzle remain scattered, but when the strain reaches a preset value (e.g., key ε = 25%), the complete image is reconstructed (movie S4).

## DISCUSSION

We have proposed a metamaterial to mimic unique mechanical behaviors of polydomain materials, i.e., inhomogeneous deformations, soft and semisoft elasticity, and polydomain-monodomain transition of nematic LCEs upon tension, providing physical insights into the multi-scale mechanics of polydomain materials. Through direct observation of lattice deformation, we have revealed previously elusive microscopic mechanisms governing these complex materials, while quantitatively reproducing various mechanical behaviors of LCEs. We developed a homogenized model based on micropolar theory to quantitatively predict the anomalous mechanical behaviors in polydomain materials. Our micropolar continuum model, experiments, and simulations show remarkably consistency on diverse problems.

This metamaterial has powerful programmable capabilities and can exhibit intriguing mechanical and information properties such as unusual zigzag deformations, mechanical transmission of patterning binary information, and strain-triggered decoders. We expect that this concept can be implemented into other domain-based systems such as magnetic materials for potential functions and applications. Our results provide a paradigm for studying mechanical polydomain systems through bridging molecular mechanisms and macroscopic properties via metastructures as analogs, opening opportunities for understanding fundamental mechanisms of bulk materials underling their complex microstructural domains and also expanding metamaterial designs that surpass the intrinsic material limits.

## METHODS

### The symmetry decomposition of the constitutive matrices

For a 3D chiral medium, the tensor H contains the chirality information. However, the tensor H must be zero in 2D case. Besides, the constitutive matrices C and D can be decomposed into several parts (*51*). According to the symmetry of the square lattice structure, the matrices should satisfy twofold and fourfold symmetry. Thus, the C and D are divided as

$$C=C^{\text{hemi}}+C^{\text{twofold}}+C^{\text{fourfold}}$$ (6)

$$D=D^{\text{hemi}}+D^{\text{twofold}}$$ (7)

where the superscripts hemi, fourfold, and twofold represent hemitropic (isotropic chiral), fourfold symmetry, and twofold symmetry components, respectively. Specifically, one obtains

$$C^{\text{hemi}}=\left[\begin{array}{cccc} \lambda +2\mu & \lambda & A & -A\\ \lambda & \lambda +2\mu & A & -A\\ A & A & \mu +k & \mu -k\\ -A & -A & \mu -k & \mu +k \end{array}\right]$$ (8)

$$\begin{array}{c} C^{\text{twofold}}=\left[\begin{array}{cccc} \beta_{1}+\beta_{2} & 0 & C_{1} & C_{2}\\ 0 & -\left(\beta_{1}+\beta_{2}\right) & C_{2} & C_{1}\\ C_{1} & C_{2} & \beta_{1}-\beta_{2} & 0\\ C_{2} & C_{1} & 0 & -\left(\beta_{1}-\beta_{2}\right) \end{array}\right] \end{array}$$ (9)

$$C^{\text{fourfold}}=\left[\begin{array}{cccc} \alpha & -\alpha & B & B\\ -\alpha & \alpha & -B & -B\\ B & -B & -\alpha & -\alpha \\ B & -B & -\alpha & -\alpha \end{array}\right]$$ (10)

$$D^{\text{hemi}}=\left[\begin{array}{cc} \gamma & 0\\ 0 & \gamma \end{array}\right]$$ (11)

$$D^{\text{twofold}}=\left[\begin{array}{cc} \gamma_{1} & \gamma_{2}\\ \gamma_{2} & -\gamma_{1} \end{array}\right]$$ (12)

Thirteen material parameters (λ, μ, A, k, $\beta_{1}$, $\beta_{2}$, $C_{1}$, $C_{2}$, α, B, γ, $\gamma_{1}$, and $\gamma_{2}$) are introduced, where λ, μ, k, and γ are the isotropic micropolar material constants. The constants A, B, $C_{1}$, and $C_{2}$ are the chiral parameters, which change their signs when the chirality is opposite, and $A=B=C_{1}=C_{2}=0$ implies no chirality exists. By solving these parameters, one can predict the mechanical properties of metamaterials (such as elastic modulus and Poisson’s ratio) and anomalous deformations (Supplementary Materials).

### Numerical method

We performed finite element simulations in commercial software Abaqus based on parameters obtained from experiments. Because deformations of the metastructures can be large (up to 40% tensile strain), the widely used hyperelastic neo-Hookean (nHk) constitutive is applied for soft beams, while linear elastic constitution is used for the rigid block. The elastic strain energy density function of the nHk model is defined as

$$\Psi_{\text{nHk}}=C_{10}\left(I_{1}-3\right)+\frac{1}{D_{1}}{\left(J-1\right)}^{2}$$ (13)

in which the material parameters are defined as $C_{10}=E\;/\;4\left(1+\nu \right)$ and $D_{1}=6\left(1-2\nu \right)\;/\;E$. The volume change reads J=det(F), where F denotes the deformation gradient tensor. The first strain invariant is given by $I_{1}=\text{tr}\left(F^{T}\cdot F\right)$. We coupled two-node linear beam elements (B31) for the soft beams and thin shell elements (S4R) for the rigid block by using a “tie” constraint at the connecting point. Mesh convergence was carefully examined. To ensure the nonlinear calculation of large deformations, we applied the dynamic relaxation method through introducing velocity-dependent damping ($C_{\text{dmp}}$) and artificial inertial (M) terms into the static equilibrium equation [$R\left(U,\lambda_{\text{dmp}}\right)=0$], leading to

$$M\frac{d^{2}U}{dt^{2}}+C_{\text{dmp}}\frac{dU}{dt}+R\left(U,\lambda_{\text{dmp}}\right)=0$$ (14)

where R, U, and $\lambda_{\text{dmp}}$ represent the residual force, unknown variables, and incremental loading parameter, respectively. Since we focus on the stable status (quasi-static), viscous energy dissipation remains quite small such that the artificial damping does not notably perturb the solution.

### Experimental method for polydomain metastructures

It is a common and crucial problem to ensure a tight bond between different phases in composite materials and structures (*52*–*54*). To achieve desired functionalities, our metamaterial sheets are required to withstand substantial tensile strain without incurring structural damage. However, the bond between TPU and PLA is not sufficiently reliable. Therefore, it is essential to design some mechanical structures to enhance the connection between these two materials without affecting their functions. We adopted two design strategies: increasing the contact area between the two materials and using mechanical interlocking. As shown in fig. S6, the arrow-shaped and trapezoidal embedded structures were designed.

## Supplementary Materials

### The PDF file includes:

Supplementary Text Figs. S1 to S6 Legends for movies S1 to S4 References
