# Supplementary material for: Programmable mechanical metastructures modeling polydomain materials
Source: Sci Adv. 2025 Oct 3;11(40):eadz9811. doi: 10.1126/sciadv.adz9811 (PMC13155561; doi:10.1126/sciadv.adz9811)
Supplement: Supplementary file 1 — Supplementary Text Figs. S1 to S6 Legends for movies S1 to S4 References [file sciadv.adz9811_sm.pdf]

Science Advances
AAAS

Supplementary Materials for

Programmable mechanical metastructures modeling polydomain materials

Yifan Yang *et al.*

Corresponding author: Fan Xu, fanxu@fudan.edu.cn

Sci. Adv. **11**, eadz9811 (2025)
DOI: 10.1126/sciadv.adz9811

The PDF file includes:

Supplementary Text
Figs. S1 to S6
Legends for movies S1 to S4
References

Other Supplementary Material for this manuscript includes the following:

Movies S1 to S4

Here we provide more details about the theoretical derivations of micropolar homogenization theory, numerical methods and resolution techniques, and discussions on the mechanical characteristics of pseudo liquid crystal metamaterials (PLCM).

I. GEOMETRY OF PLCM LATTICE

As shown in Fig. S1, the square lattice with side length L consists of a rectangular block and four soft beams. Since the rectangular block is much stiffer than the beams, it is reasonable to neglect the deformation of the rectangular block, and thus the block can be treated as a rigid body. Each beam is connected to both the corners of the rectangular block and the vertices of the square lattice. The symbols a and b represent the side lengths of the rectangular block. The lengths of the beams are denoted by L_1 and L_2. Here, θ_0 presents the rotation angle of the rectangular block at its center point. Next, we define $\angle CAx = \varphi_1$ and $\angle BAx = \varphi_2$. The directions of the beams are represented by Θ_1 and Θ_2. The geometric relations read

$$\begin{cases} L_1 = \sqrt{(L/2 + x_1)^2 + (L/2 + y_1)^2} \\ L_2 = \sqrt{(L/2 + x_2)^2 + (L/2 - y_2)^2} \\ r = \sqrt{a^2/4 + b^2/4} \\ \varphi_1 = \theta_0 + 3\pi/2 - \arctan(a/b) \\ \varphi_2 = \theta_0 + \pi/2 + \arctan(a/b) \\ \Theta_1 = \arctan[(y_1 + L/2)/(x_1 + L/2)] \\ \Theta_2 = \pi - \arctan[(L/2 - y_2)/(x_2 + L/2)] \end{cases}, \tag{S1}$$

where (x_1, y_1) and (x_2, y_2) are the coordinates of points C and B, respectively.

II. HOMOGENIZATION FOR PLCM

We apply strain energy to homogenize the lattice [36, 51]. Let $\mathbf{u}_{p,q} = [u_0\ v_0\ \phi_0]^{\mathrm{T}}$ denote the motion of the bottom-left corner in the lattice marked as (p, q). Referring to Fig. S1(b), strain energy of the square lattice (p, q) can be expressed as

$$\psi_{p,q} = \psi(\mathbf{u}_{p,q}, \mathbf{u}_{p+1,q}, \mathbf{u}_{p,q+1}, \mathbf{u}_{p+1,q+1}), \tag{S2}$$

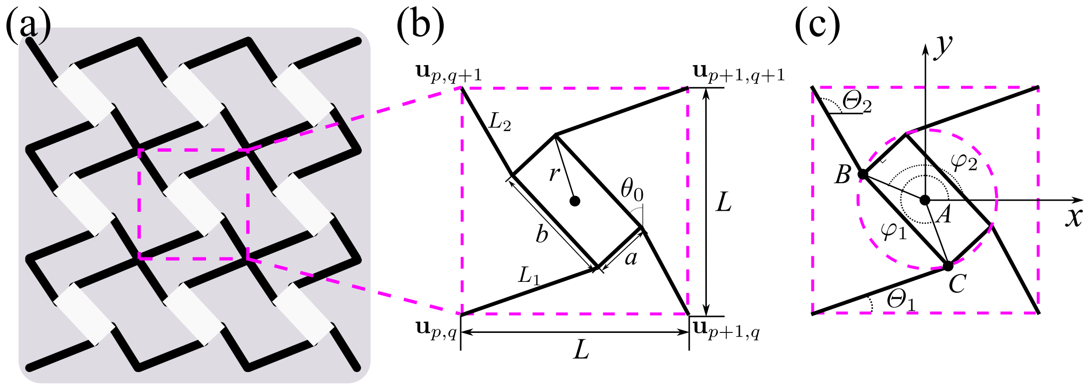

FIG. S1. Geometry of a square lattice.

where

$$\begin{cases} \mathbf{u}_{p+1,q} = \mathbf{u}_{p,q} + L[u_{,x} \ \ v_{,x} \ \ \phi_{,x}]^{\mathrm{T}} \\ \mathbf{u}_{p,q+1} = \mathbf{u}_{p,q} + L[u_{,y} \ \ v_{,y} \ \ \phi_{,y}]^{\mathrm{T}} \\ \mathbf{u}_{p+1,q+1} = \mathbf{u}_{p,q} + L[u_{,x} + u_{,y} \ \ v_{,x} + v_{,y} \ \ \phi_{,x} + \phi_{,y}]^{\mathrm{T}} \end{cases}. \tag{S3}$$

Since the rectangular block is assumed to be a rigid body, we consider only the strain energy of the four beams. As shown in Fig. S1(c), let the vector $\mathbf{u}_A = [u_A \ \ v_A \ \ \phi_A]^{\mathrm{T}}$ denote the displacement and rotation DOFs of the center of the rigid block, one obtains

$$\mathbf{u}_A = \mathbf{u}_{p,q} + \frac{L}{2}[u_{,x} + u_{,y} \ \ v_{,x} + v_{,y} \ \ \phi_{,x} + \phi_{,y}]^{\mathrm{T}}. \tag{S4}$$

The displacement and rotation of the beam ends (B, C) can be given by

$$\mathbf{u}_C = \mathbf{T}_{3\times3}(\varphi_1)\,\mathbf{u}_A, \ \ \mathbf{u}_B = \mathbf{T}_{3\times3}(\varphi_2)\,\mathbf{u}_A, \tag{S5}$$

where the transformation matrix under small deformation hypothesis is expressed as [55]

$$\mathbf{T}_{3\times3}(\varphi) = \begin{bmatrix} 1 & 0 & -r\sin\varphi \\ 0 & 1 & r\cos\varphi \\ 0 & 0 & 1 \end{bmatrix}. \tag{S6}$$

A rotation matrix $\mathbf{R}_{3\times3}$ is used to convert the local coordinates of the beam to global coordinates, defined as

$$\mathbf{R}_{3\times3} = \begin{bmatrix} \cos(-\Theta_i) & -\sin(-\Theta_i) & 0 \\ \sin(-\Theta_i) & \cos(-\Theta_i) & 0 \\ 0 & 0 & 1 \end{bmatrix}. \tag{S7}$$

In 2D Euler-Bernoulli beam theory, the stiffness matrix of the beam in its local coordinate system is expressed as

$$\hat{\mathbf{K}} = \frac{E_s t^2}{6l^3} \begin{bmatrix} 6l^2 & 0 & 0 & -6l^2 & 0 & 0 \\ 0 & 6t^2 & 3t^2 l & 0 & -6t^2 & 3t^2 l \\ 0 & 3t^2 l & 2t^2 l^2 & 0 & -3t^2 l & t^2 l^2 \\ -6l^2 & 0 & 0 & 6l^2 & 0 & 0 \\ 0 & -6t^2 & -3t^2 l & 0 & 6t^2 & -3t^2 l \\ 0 & 3t^2 l & t^2 l^2 & 0 & -3t^2 l & 2t^2 l^2 \end{bmatrix}, \tag{S8}$$

where E_s denotes elastic modulus of the beam. Then the stiffness matrix of the beam in the global coordinate can be calculated by

$$\mathbf{K} = \mathbf{T}_{6\times6}^{\mathrm{T}} \mathbf{R}_{6\times6}^{\mathrm{T}} \hat{\mathbf{K}} \mathbf{R}_{6\times6} \mathbf{T}_{6\times6}, \tag{S9}$$

where

$$\mathbf{T}_{6\times6} = \begin{bmatrix} \mathbf{I}_{3\times3} & 0 \\ 0 & \mathbf{T}_{3\times3}(\varphi_i) \end{bmatrix}, \tag{S10}$$

$$\mathbf{R}_{6\times6} = \begin{bmatrix} \mathbf{R}_{3\times3} & 0 \\ 0 & \mathbf{R}_{3\times3} \end{bmatrix}. \tag{S11}$$

Note that for beam L_1, $\mathbf{R}_{3\times3}^1 = \mathbf{R}_{3\times3}(\Theta_1)$, while for beam L_2, $\mathbf{R}_{3\times3}^2 = \mathbf{R}_{3\times3}(\pi + \Theta_2)$. Furthermore, the strain energy of each beam is expressed as

$$\begin{aligned} W_1 &= \frac{1}{2} \{\mathbf{u}_{p,q}\ \mathbf{u}_A\}^{\mathrm{T}} \mathbf{K}|_{l=L_1} \{\mathbf{u}_{p,q}\ \mathbf{u}_A\}, \\ W_2 &= \frac{1}{2} \{\mathbf{u}_{p,q+1}\ \mathbf{u}_A\}^{\mathrm{T}} \mathbf{K}|_{l=L_2} \{\mathbf{u}_{p,q+1}\ \mathbf{u}_A\}. \end{aligned} \tag{S12}$$

Due to symmetry of the lattice, the strain energy density of the lattice (p, q) reads

$$\psi_{p,q} = 2(W_1 + W_2)/L^2. \tag{S13}$$

For 2D deformation, the tensor $H_{\alpha\beta\gamma}$ in Eq. (1) in the main text vanishes, and consequently the equation can be simplified as

$$\psi = \frac{1}{2} C_{\alpha\beta\gamma\rho} \varepsilon_{\alpha\beta} \varepsilon_{\gamma\rho} + \frac{1}{2} D_{\alpha\beta} \kappa_{\alpha3} \kappa_{\beta3}. \tag{S14}$$

The expressions for $C_{\alpha\beta\gamma\rho}$ and $D_{\alpha\beta}$ can be derived as

$$C_{\alpha\beta\gamma\rho} = \frac{\partial^2 \psi}{\partial \varepsilon_{\alpha\beta} \partial \varepsilon_{\gamma\rho}}, \quad D_{\alpha\beta} = \frac{\partial^2 \psi}{\partial \kappa_{\alpha3} \partial \kappa_{\beta3}}, \tag{S15}$$

where their matrix forms are given by [51]

$$\mathbf{C} = \begin{bmatrix} \alpha+\beta_1+\beta_2+\lambda+2\mu & \lambda-\alpha & A+B+C_1 & B-A+C_2 \\ \lambda-\alpha & \alpha-\beta_1-\beta_2+\lambda+2\mu & A-B+C_2 & C_1-B-A \\ A+B+C_1 & A-B+C_2 & \beta_1-\alpha-\beta_2+k+\mu & \mu-k-\alpha \\ B-A+C_2 & C_1-B-A & \mu-k-\alpha & \beta_2-\beta_1-\alpha+k+\mu \end{bmatrix},$$

$$\mathbf{D} = \begin{bmatrix} \gamma_1+\gamma & \gamma_2 \\ \gamma_2 & \gamma-\gamma_1 \end{bmatrix}. \tag{S16}$$

Through the equivalence of the energy expressions in Eq. (S13) and (S14), all the material parameters in Eq. (S16) can be determined,

$$\alpha = \frac{E_s L^2 t}{8A_c}\left(\frac{\sin(2\Theta_2)\left({l_2}^2-t^2\right)}{{l_2}^3} - \frac{\sin(2\Theta_1)\left({l_1}^2-t^2\right)}{{l_1}^3}\right), \tag{S17}$$

$$\lambda = -\alpha, \tag{S18}$$

$$\beta_1 = 0, \tag{S19}$$

$$\beta_2 = \frac{E_s L^2 t\left(\cos(2\Theta_2)\,{l_1}^3{l_2}^2 - \cos(2\Theta_2)\,{l_1}^3t^2 + \cos(2\Theta_1)\,{l_1}^2{l_2}^3 - \cos(2\Theta_1)\,{l_2}^3t^2\right)}{4A_c{l_1}^3{l_2}^3}, \tag{S20}$$

$$\mu = \frac{E_s L^2 t\,(l_1+l_2)\left({l_1}^2{l_2}^2 + {l_1}^2t^2 - l_1l_2t^2 + {l_2}^2t^2\right)}{8A_c{l_1}^3{l_2}^3}, \tag{S21}$$

$$\begin{aligned} k = &\frac{E_s L^2 t\left({l_1}^3{l_2}^2\sin(2\Theta_2) - {l_1}^3t^2\sin(2\Theta_2) - {l_1}^2{l_2}^3\sin(2\Theta_1) + {l_2}^3t^2\sin(2\Theta_1)\right)}{8A_c{l_1}^3{l_2}^3} \\ &+ \frac{E_s L^2 t\left({l_1}^2{l_2}^3 + {l_1}^3{l_2}^2 + {l_1}^3t^2 + {l_2}^3t^2\right)}{8A_c{l_1}^3{l_2}^3} \end{aligned}, \tag{S22}$$

$$A = -\frac{E_s L^2 t\left(-\cos(2\Theta_2)\,{l_1}^3{l_2}^2 + \cos(2\Theta_2)\,{l_1}^3t^2 + \cos(2\Theta_1)\,{l_1}^2{l_2}^3 - \cos(2\Theta_1)\,{l_2}^3t^2\right)}{8A_c{l_1}^3{l_2}^3}, \tag{S23}$$

$$B = -A \tag{S24}$$

$$C_1 = \frac{E_s L^2 t}{4A_c}\left(\frac{\sin(2\Theta_1)\left({l_1}^2-t^2\right)}{{l_1}^3} + \frac{\sin(2\Theta_2)\left({l_2}^2-t^2\right)}{{l_2}^3}\right), \tag{S25}$$

$$C_2 = -\frac{E_s L^2 t\,(l_1-l_2)\left({l_1}^2{l_2}^2 + {l_1}^2t^2 + l_1l_2t^2 + {l_2}^2t^2\right)}{4A_c{l_1}^3{l_2}^3}, \tag{S26}$$

$$\begin{aligned}\gamma = &\frac{E_sL^2t\left(3{l_1}^2{l_2}^3r^2+3{l_1}^3{l_2}^2r^2+2{l_1}^2{l_2}^3t^2+8{l_1}^3{l_2}^2t^2+3{l_1}^3r^2t^2\right)}{12A_c{l_1}^3{l_2}^3}\\&+\frac{E_sL^2t\left(3{l_2}^3r^2t^2-3{l_1}^2{l_2}^3r^2\cos\left(2\Theta_1-2\phi_1\right)-3{l_1}^3{l_2}^2r^2\cos\left(2\Theta_2-2\phi_2\right)\right)}{12A_c{l_1}^3{l_2}^3}\\&+\frac{E_sL^2t\left(3{l_2}^3r^2t^2\cos\left(2\Theta_1-2\phi_1\right)+3{l_1}^3r^2t^2\cos\left(2\Theta_2-2\phi_2\right)\right)}{12A_c{l_1}^3{l_2}^3}\\&+\frac{E_sL^2t\left(12{l_1}^3l_2rt^2\cos\left(\Theta_2-\phi_2\right)-6l_1\,{l_2}^3rt^2\cos\left(\Theta_1-\phi_1\right)\right)}{12A_c{l_1}^3{l_2}^3}\end{aligned}, \tag{S27}$$

$$\gamma_1 = -\frac{E_sL^2t^3\left(l_2+r\cos\left(\Theta_2-\phi_2\right)\right)}{2A_c{l_2}^2}, \tag{S28}$$

$$\begin{aligned}\gamma_2 = &\frac{E_sL^2t\left(3{l_1}^2{l_2}^3r^2+3{l_1}^3{l_2}^2r^2+2{l_1}^2{l_2}^3t^2+4{l_1}^3{l_2}^2t^2+3{l_1}^3r^2t^2\right)}{12A_c{l_1}^3{l_2}^3}\\&+\frac{E_sL^2t\left(3{l_2}^3r^2t^2-3{l_1}^2{l_2}^3r^2\cos\left(2\Theta_1-2\phi_1\right)-3{l_1}^3{l_2}^2r^2\cos\left(2\Theta_2-2\phi_2\right)\right)}{12A_c{l_1}^3{l_2}^3}\\&+\frac{E_sL^2t\left(3{l_2}^3r^2t^2\cos\left(2\Theta_1-2\phi_1\right)+3{l_1}^3r^2t^2\cos\left(2\Theta_2-2\phi_2\right)\right)}{12A_c{l_1}^3{l_2}^3}\\&+\frac{E_sL^2t\left(12{l_1}^3l_2rt^2\cos\left(\Theta_2-\phi_2\right)-6l_1{l_2}^3rt^2\cos\left(\Theta_1-\phi_1\right)\right)}{12A_c{l_1}^3{l_2}^3}\end{aligned}. \tag{S29}$$

For example, the effective material parameters of the lattice with $L = 15$ mm, $ab/L^2 = 0.225$, $\eta = 2.5$, $t = L/10$ and $E_s = 9.5$ MPa are plotted in Fig. S2(a) and (b). It can be observed that $B = -A$, which is associated with the symmetry of the lattice. Note that when $\theta_0 = 0°$, the chiral parameters become zero and consequently, the chirality of the lattice disappears. Moreover, when the sign of θ_0 reverses, the signs of the chiral parameters change accordingly, demonstrating that these parameters effectively characterize the lattice's chiral properties.

To further investigate the mechanical properties of the PLCM lattice, the most fundamental parameters, namely the effective elastic modulus and Poisson's ratio are calculated based on the micropolar homogenization model. Here, all stress components are assumed to be zero except for σ_{22}. Consequently, all strain components can be obtained through the constitutive relationship. By defining $\nu = -\varepsilon_{11}/\varepsilon_{22}$ and $E = \sigma_{22}/\varepsilon_{22}$, the dimensionless effective elastic modulus and Poisson's ratio under various geometric parameters are depicted as the solid curves in Fig. S2(c) and (d). For comparison, lattices with the same parameters are calculated. The boundary conditions are illustrated in the schematic inset in Fig. S2(c). Both the theory and simulation results indicate that a smaller aspect ratio of the rigid block η and a smaller central rotation angle θ_0 lead to a larger elastic modulus.

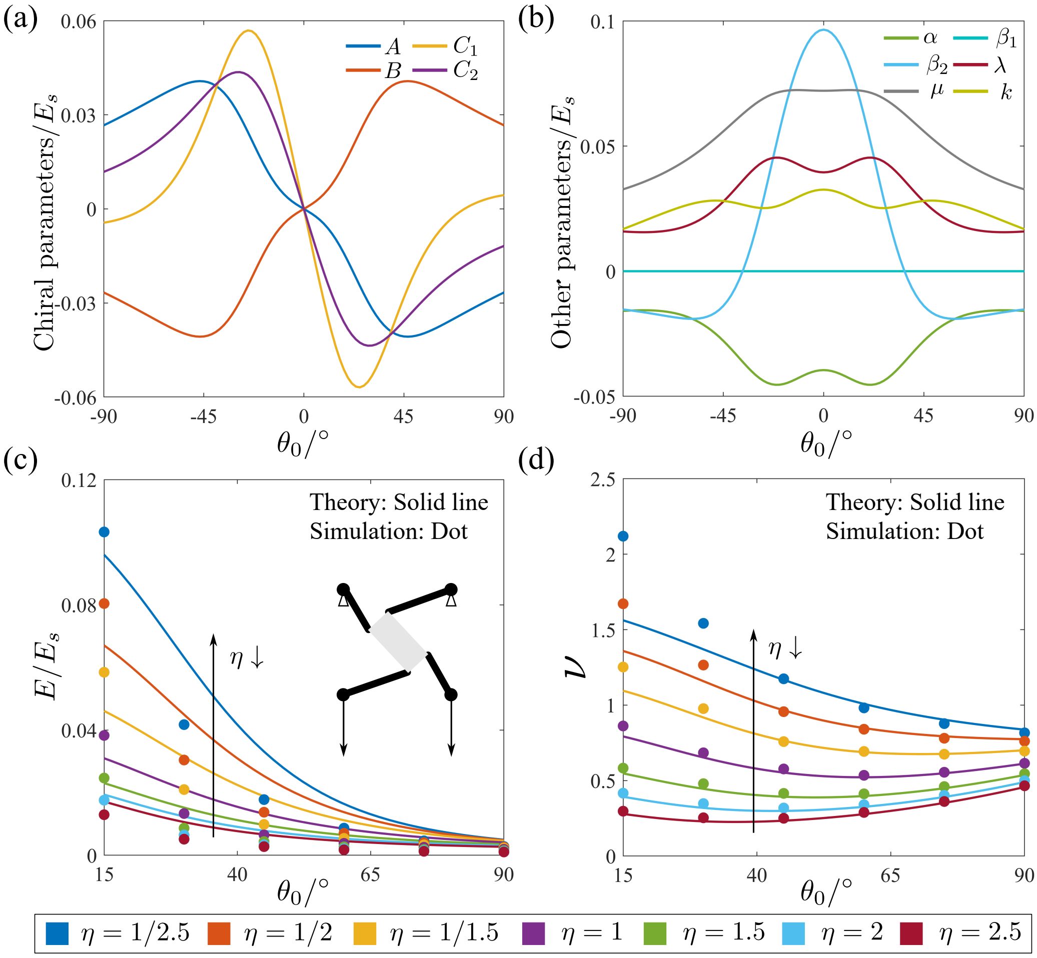

FIG. S2. Mechanical properties in micropolar homogenization theory. (a)-(b) Some effective material parameters ($L = 15$ mm, $ab/L^2 = 0.225$, $\eta = 2.5$, $t = L/10$ and $E_s = 9.5$ MPa). (c)-(d) Young's modulus and Poisson's ratio of a single lattice.

This occurs because under stretching, the beams within the lattice with parameters $\eta < 1$ and small θ_0 undergo more elongation rather than bending and rigid-body motion, making these lattices appear "harder". As shown in Fig. S2(d), Poisson's ratios of the lattices are always positive, and the lattices with smaller η have larger Poisson's ratios. Our theoretical predictions are consistent with computations.

III. UNIAXIAL TENSION

For the static case, the equilibrium equations of a planar micropolar elastomer read [36]

$$\begin{aligned} &\sigma_{\beta\alpha,\beta} = 0, \\ &m_{\alpha 3,\alpha} + e_{3\alpha\beta}\sigma_{\alpha\beta} = 0. \end{aligned} \tag{S30}$$

By combining geometric equations $\varepsilon_{\alpha\beta} = u_{\beta,\alpha} + e_{3\beta\alpha}\phi_3$, $\kappa_{\alpha 3} = \phi_{3,\alpha}$ and neglecting the subscript 3, the vectorization of Cauchy stress and moment can be written as

$$\begin{bmatrix} \sigma_{11} \\ \sigma_{22} \\ \sigma_{12} \\ \sigma_{21} \end{bmatrix} = \mathbf{C} \begin{bmatrix} \varepsilon_{11} \\ \varepsilon_{22} \\ \varepsilon_{12} \\ \varepsilon_{21} \end{bmatrix} = \mathbf{C} \begin{bmatrix} u_{,1} \\ v_{,2} \\ v_{,1} - \phi \\ u_{,2} + \phi \end{bmatrix}, \tag{S31}$$

$$\begin{bmatrix} m_1 \\ m_2 \end{bmatrix} = \mathbf{D} \begin{bmatrix} \phi_{,1} \\ \phi_{,2} \end{bmatrix}. \tag{S32}$$

Here, we consider uniaxial stretching in the y direction, where the problem is infinite in the x direction, and the field quantities depend only on y. The strains can be expressed as ordinary derivatives with respect to y and are simplified as

$$\varepsilon_{xx} = 0,\ \varepsilon_{yy} = v',\ \varepsilon_{xy} = -\phi,\ \varepsilon_{yx} = u' + \phi. \tag{S33}$$

By substituting Eqs. (S31)-(S33) into Eq. (S30), one obtains the governing equations as

$$\begin{cases} 0 = (\beta_2 - \beta_1 - \alpha + k + \mu)\, u'' - (C_1 - B - A)\, v'' + (-\beta_1 + \beta_2 + 2k)\, \phi' \\ 0 = -(C_1 - B - A)\, u'' + (\alpha - \beta_1 - \beta_2 + \lambda + 2\mu)\, v'' + (2A - C_1 + C_2)\, \phi' \\ 0 = (\gamma - \gamma_1)\, \phi'' + (\beta_1 - \beta_2 - 2k)\, u' + (C_1 - C_2 - 2A)\, v' - 4k\phi \end{cases}. \tag{S34}$$

The above equations form a classical boundary value problem for ordinary differential equations, which can be solved using MATLAB. The boundary conditions for clamped ends are specified as $u(0) = 0$, $v(0) = 0$, $\phi(0) = 0$, $u(L_0) = 0$, $v(L_0) = \Delta v$, and $\phi(L_0) = 0$, where Δv represents the displacement load, and L_0 denotes the total length. For freely rotating ends, the boundary conditions are given by $u(0) = 0$, $v(0) = 0$, $u''(0) = 0$, $u(L_0) = 0$, $v(L_0) = \Delta v$, and $u''(L_0) = 0$. To solve uniaxial tension problem with multiple domain distributions (as shown in Fig. 4 in the main text), the material parameters $\mathbf{C}$ and $\mathbf{D}$ can be piecewise defined according to the domain distributions during the solution process.

IV. EFFECTS OF PARAMETERS ON PLCM

The rotation of liquid crystal directors leads to novel mechanical behaviors of LCE films. Similarly, the rotation of the PLCM lattice under stretch is caused by the rotation of the rigid block. The underlying mechanism results from the anisotropic elastic energy distribution within each unit cell, where the rigid block acts as a director analog. Here, the stretch-rotation behavior of a single lattice is first explored using the commercial software Abaqus [56]. We consider a single lattice with different geometric parameters ($\theta_0 = 0° \sim 90°$, $\eta = 1/3 \sim 3$). To clearly observe the rotation of the whole lattice, the top and bottom ends of the lattice are fixed by two rigid rods with simple support conditions at their midpoints. Then a displacement load is applied at the midpoint of the bottom rod, leading the rods to rotate by φ_r (as shown in Fig. S3(a)). Based on finite element simulations, computed heatmaps illustrating φ_r with respect to various θ_0, η and strain ε are given in Fig. S3(b)-(d). The rotation behavior of the lattice arises from the competition between elastic strain energy minimization and geometric constraints imposed by the rigid block orientation. Therefore, this behavior is governed by the initial orientation and the aspect ratio of the rigid block. Similar to the liquid crystal rod molecule, the aspect ratio of the rigid block η represents the anisotropy of the lattice. When $\eta \sim 1$, the stretch-rotation effect is minimal, while as η deviates further from 1 (*i.e.*, the rigid block becomes more elongated), the rotation effect becomes more pronounced. For $\eta < 1$, when θ_0 is relatively small (e.g., 15°, 30° and 45°), the rotation angle φ_r remains negative (clockwise rotation) or change from positive to negative with the increasing of strain ε. Additionally, with the increase of η, different initial orientations of rigid block θ_0 may lead to different rotation tendencies of the whole lattice. Specifically, when θ_0 is roughly in the range of $0° \sim 60°$, φ_r increases monotonically with η. When θ_0 is generally between 75° and 90°, φ_r consistently decreases as η rises because the rigid block is already near-aligned with the stretching direction, causing resistance to further rotation. Comparing Fig. S3(b), (c) and (d), one can see that the rotation angle φ_r becomes larger when the strain increases.

For multiple lattices in metamaterial sheets, the arrangement of lattices introduces additional mechanical responses, as neighboring lattices impose boundary constraints that change the local loads and rotation kinematics compared to isolated lattices. The collective behavior emerges from the interplay between single lattice rotation tendencies and

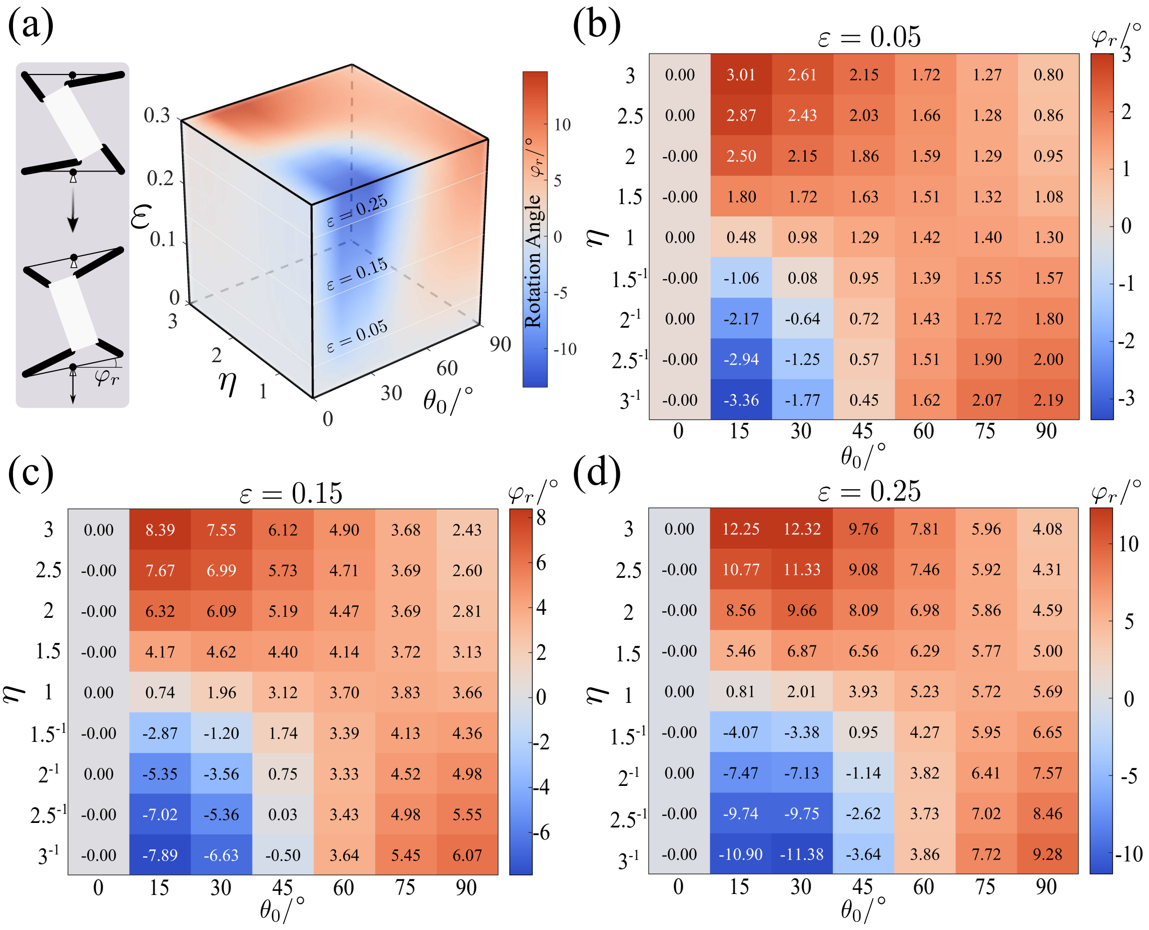

FIG. S3. Rotational deformation tests of lattices. (a) Schematic of stretched PLCM lattice with simply supported boundary conditions and the 3D rotational performance map by simulation. (b)-(d) Heat maps of PLCM lattice rotation with respect to parameters η and θ_0.

inter-lattice mechanical coupling, creating a mesoscale analog to polydomain interactions in liquid crystal elastomers. When considering a fixed number of lattices arranged into a rectangular sheet, the ratio of rows to columns influences the behavior of the sheet under similar loading conditions. In Fig. S4(a), 100 identical lattices are arranged in configurations of $2 \times 50, 4 \times 25, 5 \times 20, 10 \times 10, 20 \times 5, 25 \times 4, 50 \times 2$ and 100×1. The FEM simulation results show that for the sheet with a smaller row/column ratio, the change of rotation under tension is slight since the small aspect ratio induces strong boundary constraints that restrict rotational freedom. Specifically, the rotation angle reaches its peak when the sheet is square. Then the rotation angle decreases as the row/column ratio increases. Therefore, the square configuration optimizes the balance between stretching loads and rotational freedom, allowing each lattice to rotate with minimal constraints from neighbors. In addition, a larg-

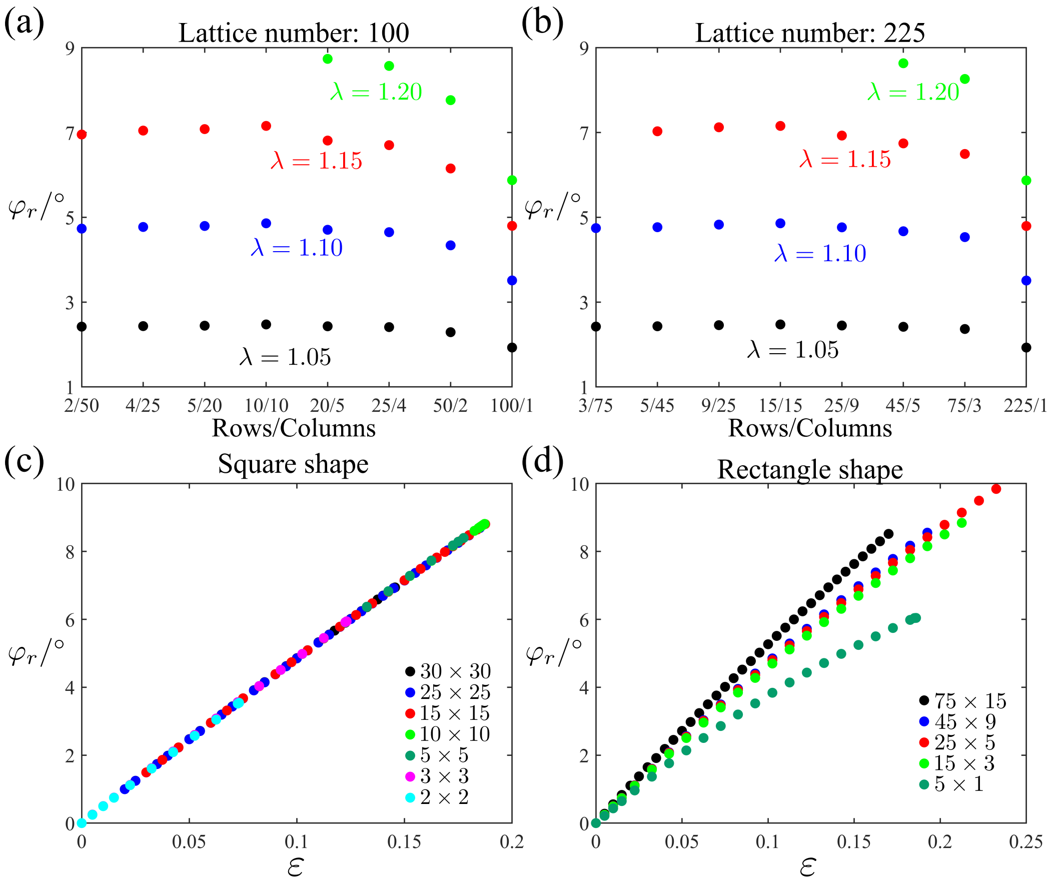

FIG. S4. The shape and size effects of the pseudo liquid crystal metamaterial. The parameters of example lattice are: $L = 15$ mm, $ab/L^2 = 0.225$, $\eta = 2.5$ and $t = L/10$. In (a) and (b), metamaterial sheets with various ratios of row/column and fixed numbers of lattices are simulated. In (c)-(d), metamaterial sheets with fixed ratios row/column and various numbers of lattices are simulated.

er strain leads to increased rotation angles. However, it is notable that slender rectangular sheets exhibit better stretching capability. To verify these trends, another set of similar sheets with 225 lattices is simulated in Fig. S4(b), yielding similar conclusions.

Size effect would be another key factor. We consider a series of fixed-size square sheets consisting of identical lattices. When the number of lattices gradually increases from 4 to 900, as shown in Fig. S4(c), the relation between φ_r and ε remains almost unchanged, implying that the size effect on the stretch-rotation behavior of the sheets can be neglected for square configurations, where symmetric boundary conditions preserve scale-independence. However, when the row/column ratio is set to be 5:1, size effect becomes non-negligible

for rectangular configurations. Figure S4(d) shows that the rotation is largest in the sheet consisting of the most lattices (75×15), while the sheet with the fewest lattices (5×1) exhibits the smallest rotation due to the dominance of boundary constraints in slender systems. As the lattice density increases, the rotation angle increases and converges to the configuration of 75×15, where interior lattices can rotate freely according to their intrinsic properties and boundary effects become negligible.

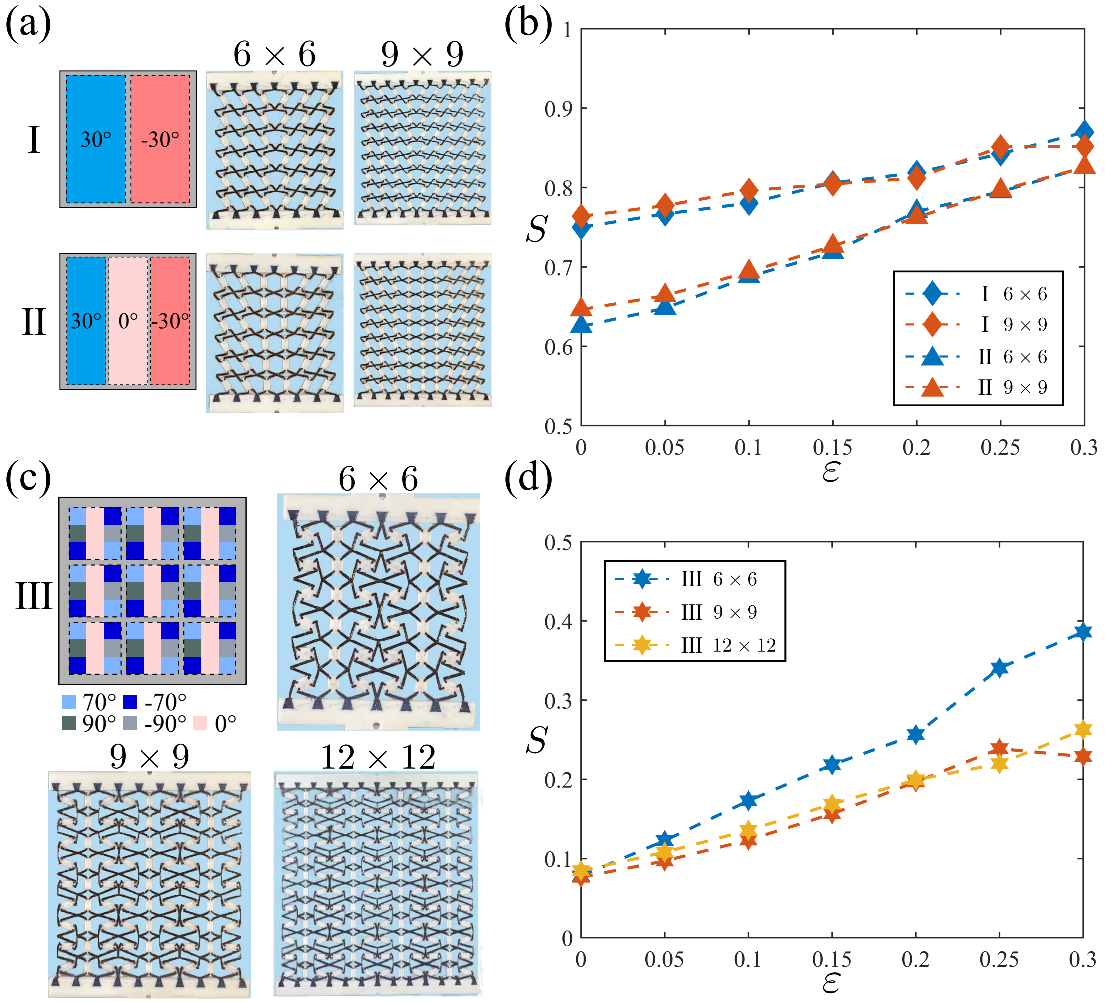

FIG. S5. Tensile test of metamaterial sheets with different polydomain configurations. (a) Two types of laterally arranged polydomain sheets and their order parameters (b). (c) The more complex polydomain sheets and their order parameters (d).

V. POLYDOMAIN-MONODOMAIN TRANSITION OF PLCM SHEETS

The polydomain-monodomain transition (P-M transition) represents a topological reorganization process where the initial polydomain structure gradually evolves into a monodomain configuration under external load. The P-M transition induced by stretching is commonly observed in polydomain materials. This transformation is governed by the competition between elastic energy minimization and orientational ordering. Here, we demonstrate that the well designed PLCM can reproduce this phenomenon, as shown in Fig. S5(a) and (c). To establish a tunable experimental framework for studying P-M transitions, Sheet I and Sheet II are, respectively, composed of two and three vertical monodomain stripes. The initial degree of order in the sheets can be designed by the arrangements of lattices with different θ_0 (e.g., $-30°, 0°$ and $30°$). The order parameter S of Sheet I and Sheet II are 0.625 and 0.75, respectively, reflecting the degree of initial structural anisotropy, which directly influences the transition process (see Fig. S5(b)). Upon stretching, the tensile force drives lattices to reorient and align with each other, reducing the total elastic energy in the system. This energy-driven process follows the micropolar continuum mechanics principles, where local rotational degrees of freedom couple with macroscopic deformation to achieve the degree of order in the two sheets gradually increasing and evolving towards monodomain distribution. Sheet III is composed of several substructures consisting of nine complex cellular lattices. To achieve a nearly isotropic sheet, the substructure is designed in a symmetric pattern (see Fig. S5(c)). In the tensile experiments, the order parameter S exhibits a similar tendency as Sheet I and II.

To explore the influence of lattice number on the P-M transition, Sheets I, II and III are separately produced in configurations of 6×6 and 9×9. The results show that, for Sheets I and II, the difference between size 6×6 and 9×9 remains small, indicating that the transition behavior has converged for these relatively simple domain arrangements. However, for Sheet III, the increase of order parameter S in the 6×6 sheet is quite faster than in the 9×9 sheet. This size-dependent behavior reflects the influence of boundary effects and finite-size constraints on the polydomain reorientation process. According to further refinement on 12×12 Sheet III, the configuration of 9×9 can ensure the convergence of the order parameter S (see Fig. S5(d)).

VI. REINFORCEMENT STRUCTURE DESIGNS FOR PLCM SHEETS

To improve the connection between TPU and PLA materials in the metamaterial sheets, we designed specific reinforcement structures including arrow-shaped and trapezoidal embedded geometries (Fig. S6). These structures increase the contact area and provide mechanical interlocking between the two materials.

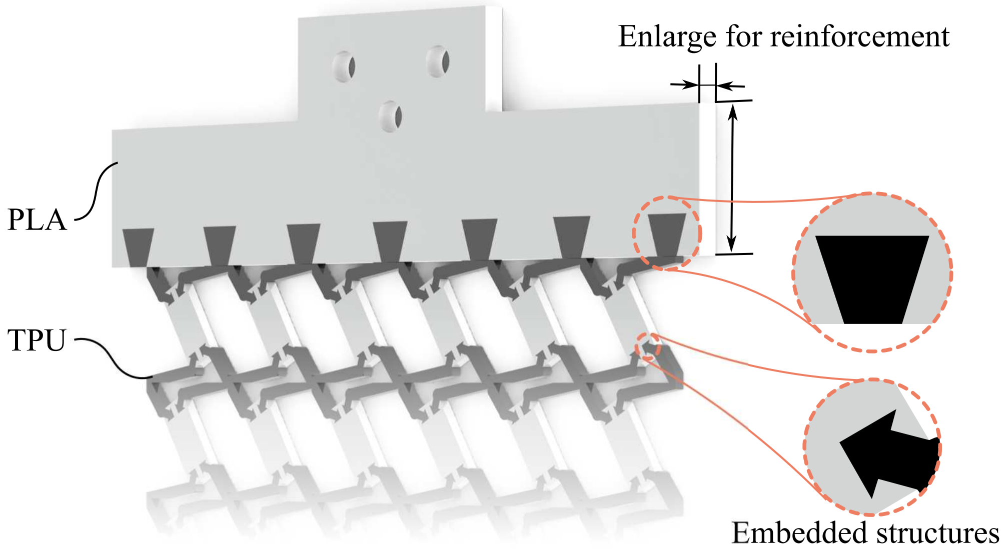

FIG. S6. Reinforcement structure designs for tensile experiments.

Movie S1. Uniaxial stretching and P-M transitions of PLCM sheets.

Movie S2. Tensile experiments of PLCM with different “crosslinking” modes.

Movie S3. Zigzag deformation of stretched PLCM strips.

Movie S4. Information encoding based on PLCM.

REFERENCES AND NOTES

1. X. Lin, J. C. Lu, Y. Shao, Y. Y. Zhang, X. Wu, J. B. Pan, L. Gao, S. Y. Zhu, K. Qian, Y. F. Zhang, D. L. Bao, L. F. Li, Y. Q. Wang, Z. L. Liu, J. T. Sun, T. Lei, C. Liu, J. O. Wang, K. Ibrahim, D. N. Leonard, W. Zhou, H. M. Guo, Y. L. Wang, S. X. Du, S. T. Pantelides, H.-J. Gao, Intrinsically patterned two-dimensional materials for selective adsorption of molecules and nanoclusters. *Nat. Mater.* **16**, 717–721 (2017).

2. Z. Li, J. Su, S.-Z. Lin, D. Liu, Y. Gao, S. Wang, H. Wei, T. Zhao, Y. Zhang, J. Cai, B. Shen, Field-free topological behavior in the magnetic domain wall of ferrimagnetic GdFeCo. *Nat. Commun.* **12**, 5604 (2021).

3. D. Liu, L. Liu, P. R. Onck, D. J. Broer, Reverse switching of surface roughness in a self-organized polydomain liquid crystal coating. *Proc. Natl. Acad. Sci. U.S.A.* **112**, 3880–3885 (2015).

4. M. E. Davis, Ordered porous materials for emerging applications. *Nature* **417**, 813–821 (2002).

5. K. Chen, L. Li, Ordered structures with functional units as a paradigm of material design. *Adv. Mater.* **31**, 1901115 (2019).

6. S. Misra, L. Li, D. Zhang, J. Jian, Z. Qi, M. Fan, H.-T. Chen, X. Zhang, H. Wang, Self-assembled ordered three-phase Au–$BaTiO_3$–ZnO vertically aligned nanocomposites achieved by a templating method. *Adv. Mater.* **31**, 1806529 (2019).

7. H. Ji, J. Wu, Z. Cai, J. Liu, D.-H. Kwon, H. Kim, A. Urban, J. K. Papp, E. Foley, Y. Tian, M. Balasubramanian, H. Kim, R. J. Clément, B. D. Mc Closkey, W. Yang, G. Ceder, Ultrahigh power and energy density in partially ordered lithium-ion cathode materials. *Nat. Energy* **5**, 213–221 (2020).

8. L. Lou, Y. Li, X. Li, H. Li, W. Li, Y. Hua, W. Xia, Z. Zhao, H. Zhang, M. Yue, X. Zhang, Directional magnetization reversal enables ultrahigh energy density in gradient nanostructures. *Adv. Mater.* **33**, 2102800 (2021).

9. M. Fan, B. Zhang, H. Wang, J. Jian, X. Sun, J. Huang, L. Li, X. Zhang, H. Wang, Self-organized epitaxial vertically aligned nanocomposites with long-range ordering enabled by substrate nanotemplating. *Adv. Mater.* **29**, 1606861 (2017).

10. M. Warner, E. M. Terentjev, *Liquid Crystal Elastomers* (Oxford Univ. Press) (2007).

11. N. A. Traugutt, D. Mistry, C. Luo, K. Yu, Q. Ge, C. M. Yakacki, Liquid-crystal-elastomer-based dissipative structures by digital light processing 3D printing. *Adv. Mater.* **32**, e2000797 (2020).

12. T. H. Ware, M. E. McConney, J. J. Wie, V. P. Tondiglia, T. J. White, Voxelated liquid crystal elastomers. *Science* **347**, 982–984 (2015).

13. I. H. Kim, S. Choi, J. Lee, J. Jung, J. Yeo, J. T. Kim, S. Ryu, S.-K. Ahn, J. Kang, P. Poulin, S. O. Kim, Human-muscle-inspired single fibre actuator with reversible percolation. *Nat. Nanotechnol.* **17**, 1198–1205 (2022).

14. M. O. Saed, W. Elmadih, A. Terentjev, D. Chronopoulos, D. Williamson, E. M. Terentjev, Impact damping and vibration attenuation in nematic liquid crystal elastomers. *Nat. Commun.* **12**, 6676 (2021).

15. H. Aharoni, Y. Xia, X. Zhang, R. D. Kamien, S. Yang, Universal inverse design of surfaces with thin nematic elastomer sheets. *Proc. Natl. Acad. Sci. U.S.A.* **115**, 7206–7211 (2018).

16. D. R. Merkel, R. K. Shaha, C. M. Yakacki, C. P. Frick, Mechanical energy dissipation in polydomain nematic liquid crystal elastomers in response to oscillating loading. *Polymer* **166**, 148–154 (2019).

17. K. M. Herbert, H. E. Fowler, Joselle M. Mc Cracken, K. R. Schlafmann, J. A. Koch, T. J. White, Synthesis and alignment of liquid crystalline elastomers. *Nat. Rev. Mater.* **7**, 23–38 (2022).

18. T. Seki, New strategies and implications for the photoalignment of liquid crystalline polymers. *Polym. J.* **46**, 751–768 (2014).

19. R. S. Kularatne, H. Kim, J. M. Boothby, T. H. Ware, Liquid crystal elastomer actuators: Synthesis, alignment, and applications. *J. Polym. Sci. Pt. B-Polym. Phys.* **55**, 395–411 (2017).

20. S.-J. Park, M. Gazzola, K. S. Park, S. Park, V. D. Santo, E. L. Blevins, J. U. Lind, P. H. Campbell, S. Dauth, A. K. Capulli, F. S. Pasqualini, S. Ahn, A. Cho, H. Yuan, B. M. Maoz, R. Vijaykumar, J.-W. Choi, K. Deisseroth, G. V. Lauder, L. Mahadevan, K. K. Parker, Phototactic guidance of a tissue-engineered soft-robotic ray. *Science* **353**, 158–162 (2016).

21. J. Hu, Z. Nie, M. Wang, Z. Liu, S. Huang, H. Yang, Springtail-inspired light-driven soft jumping robots based on liquid crystal elastomers with monolithic three-leaf panel fold structure. *Angew. Chem. Int. Ed. Engl.* **62**, e202218227 (2023).

22. S. Wu, Y. Hong, Y. Zhao, J. Yin, Y. Zhu, Caterpillar-inspired soft crawling robot with distributed programmable thermal actuation. *Sci. Adv.* **9**, eadf8014 (2023).

23. Y. Yang, S. Zhao, Z. Dai, F. Xu, Programmable wrinkling patterns of liquid crystal network bilayers on compliant substrates. *Int. J. Solids Struct.* **309**, 113206 (2025).

24. Y. Yang, Z. Dai, Y. Chen, F. Xu, Active twisting for adaptive droplet collection. *Nat. Comput. Sci.* **5**, 313–321 (2025).

25. H. E. Fowler, P. Rothemund, C. Keplinger, T. J. White, Liquid crystal elastomers with enhanced directional actuation to electric fields. *Adv. Mater.* **33**, 2103806 (2021).

26. W. Qiu, Y. Xu, F. Xu, Y. Huo, Programmable electric-field-induced bending shapes of dielectric liquid crystal elastomer sheets. *Extreme Mech. Lett.* **60**, 101982 (2023).

27. Q. He, Y. Zheng, Z. Wang, X. He, S. Cai, Anomalous inflation of a nematic balloon. *J. Mech. Phys. Solids* **142**, 104013 (2020).

28. Z. Dai, Y. Wen, Z. Chen, Y. Chen, Y. Yang, M. Gao, Y. Chen, F. Xu, Unusual stretching-twisting of liquid crystal elastomer bilayers. *J. Mech. Phys. Solids* **198**, 106066 (2025).

29. T. J. White, D. J. Broer, Programmable and adaptive mechanics with liquid crystal polymer networks and elastomers. *Nat. Mater.* **14**, 1087–1098 (2015).

30. S. C. Lamont, F. J. Vernerey, Generalized continuum theory for nematic elastomers: Non-affine motion and characteristic behavior. *J. Mech. Phys. Solids* **190**, 105718 (2024).

31. C. P. Broedersz, C. Storm, F. C. MacKintosh, Effective-medium approach for stiff polymer networks with flexible cross-links. *Phys. Rev. E* **79**, 061914 (2009).

32. A. C. Eringen, *Microcontinuum Field Theories: I. Foundations and Solids* (Springer, 1999).

33. H. Abdoul-Anziz, P. Seppecher, Strain gradient and generalized continua obtained by homogenizing frame lattices. *Math. Mech. Complex Syst.* **6**, 213–250 (2018).

34. A. Bacigalupo, L. Gambarotta, Second-gradient homogenized model for wave propagation in heterogeneous periodic media. *Int. J. Solids Struct.* **51**, 1052–1065 (2014).

35. G. Rizzi, F. Dal Corso, D. Veber, D. Bigoni, Identification of second-gradient elastic materials from planar hexagonal lattices. Part II: Mechanical characteristics and model validation. *Int. J. Solids Struct.* **176-177**, 19–35 (2019).

36. X. Liu, G. Huang, G. Hu, Chiral effect in plane isotropic micropolar elasticity and its application to chiral lattices. *J. Mech. Phys. Solids* **60**, 1907–1921 (2012).

37. X. He, Y. Zheng, Q. He, S. Cai, Uniaxial tension of a nematic elastomer with inclined mesogens. *Extreme Mech. Lett.* **40**, 100936 (2020).

38. W. Fan, Z. Wang, S. Cai, Rupture of polydomain and monodomain liquid crystal elastomer. *Int. J. Appl. Mech.* **8**, 1640001 (2016).

39. S. Clarke, E. Nishikawa, H. Finkelmann, E. Terentjev, Light-scattering study of random disorder in liquid crystalline elastomers. *Macromol. Chem. Phys.* **198**, 3485–3498 (1997).

40. N. Mottram, S. Hogan, Magnetic field-induced changes in molecular order in nematic liquid crystals. *Continuum Mech. Thermodyn.* **14**, 281–295 (2002).

41. J. Küupfer, H. Finkelmann, Liquid crystal elastomers: Influence of the orientational distribution of the crosslinks on the phase behaviour and reorientation processes. *Macromol. Chem. Phys.* **195**, 1353–1367 (1994).

42. S. Dey, D. M. Agra-Kooijman, W. Ren, P. J. Mc Mullan, A. C. Griffin, S. Kumar, Soft elasticity in main chain liquid crystal elastomers. *Crystals* **3**, 363–390 (2013).

43. L. Rezaei, G. Scalet, M. Peigney, A. Azoug, Coupling between viscoelasticity and soft elasticity in main-chain nematic liquid crystal elastomers. *J. Mech. Phys. Solids* **187**, 105612 (2024).

44. S. Kutter, E. Terentjev, Tube model for the elasticity of entangled nematic rubbers. *Eur. Phys. J. E* **6**, 221–229 (2001).

45. L.-L. Ma, W. Hu, Z.-G. Zheng, S.-B. Wu, P. Chen, Q. Li, Y.-Q. Lu, Light-activated liquid crystalline hierarchical architecture toward photonics. *Adv Opt Mater* **7**, 1900393 (2019).

46. M.-J. Gim, D. A. Beller, D. K. Yoon, Morphogenesis of liquid crystal topological defects during the nematic-smectic A phase transition. *Nat. Commun.* **8**, 15453 (2017).

47. K. Urayama, E. Kohmon, M. Kojima, T. Takigawa, Polydomain-monodomain transition of randomly disordered nematic eastomers with different cross-linking histories. *Macromolecules* **42**, 4084–4089 (2009).

48. J. Biggins, E. Terentjev, M. Warner, Semisoft elastic response of nematic elastomers to complex deformations. *Phys. Rev. E* **78**, 041704 (2008).

49. G. C. Verwey, M. Warner, Nematic elastomers cross-linked by rigid rod linkers. *Macromolecules* **30**, 4196–4204 (1997).

50. X. Xing, S. Pfahl, S. Mukhopadhyay, P. M. Goldbart, A. Zippelius, Nematic elastomers: From a microscopic model to macroscopic elasticity theory. *Phys. Rev. E* **77**, 051802 (2008).

51. Y. Chen, X. Liu, G. Hu, Micropolar modeling of planar orthotropic rectangular chiral lattices. *C. R. Mecanique* **342**, 273–283 (2014).

52. B. Zareiyan, B. Khoshnevis, Effects of interlocking on interlayer adhesion and strength of structures in 3D printing of concrete. *Autom. Constr.* **83**, 212–221 (2017).

53. H. Yang, C. Li, M. Yang, Y. Pan, Q. Yin, J. Tang, H. J. Qi, Z. Suo, Printing hydrogels and elastomers in arbitrary sequence with strong adhesion. *Adv. Funct. Mater.* **29**, 1901721 (2019).

54. A. Baldan, Adhesively-bonded joints and repairs in metallic alloys, polymers and composite materials: Adhesives, adhesion theories and surface pretreatment. *J. Mater. Sci.* **39**, 1–49 (2004).

55. A. Spadoni, M. Ruzzene, Elasto-static micropolar behavior of a chiral auxetic lattice. *J. Mech. Phys. Solids* **60**, 156–171 (2012).

56. ABAQUS, *ABAQUS Analysis Users Manual* version 6.13 (2013).
